# Maternal polycystic ovary syndrome and risk of neuropsychiatric disorders in offspring: prenatal androgen exposure or genetic confounding?

**DOI:** 10.1017/S0033291719000424

**Published:** 2020-03

**Authors:** Carolyn E. Cesta, Anna S. Öberg, Abraham Ibrahimson, Ikram Yusuf, Henrik Larsson, Catarina Almqvist, Brian M. D'Onofrio, Cynthia M. Bulik, Lorena Fernández de la Cruz, David Mataix-Cols, Mikael Landén, Mina A. Rosenqvist

**Affiliations:** 1 Department of Medical Epidemiology and Biostatistics, Karolinska Institutet, Stockholm, Sweden; 2 Centre for Pharmacoepidemiology, Department of Medicine, Karolinska Institutet, Stockholm, Sweden; 3 Department of Epidemiology, Harvard T.H. Chan School of Public Health, Boston, MA, USA; 4 School of Medical Sciences, Örebro University, Örebro, Sweden; 5 Astrid Lindgren Children's Hospital, Karolinska University Hospital, Stockholm, Sweden; 6 Department of Psychological and Brain Sciences, Indiana University, Bloomington, IN, USA; 7 Department of Psychiatry, University of North Carolina at Chapel Hill, Chapel Hill, NC, USA; 8 Department of Nutrition, University of North Carolina at Chapel Hill, Chapel Hill, NC, USA; 9 Centre for Psychiatry Research, Department of Clinical Neuroscience, Karolinska Institutet, Stockholm, Sweden; 10 Stockholm Health Care Services, Stockholm County Council, Stockholm, Sweden; 11 Institute of Neuroscience and Physiology, University of Gothenburg, Gothenburg, Sweden

**Keywords:** Androgens, epidemiology, familial confounding, neuropsychiatric disorders, polycystic ovary syndrome

## Abstract

**Background:**

Maternal polycystic ovary syndrome (PCOS) has been proposed as a model for investigating the role of prenatal androgen exposure in the development of neuropsychiatric disorders. However, women with PCOS are at higher risk of developing psychiatric conditions and previous studies are likely confounded by genetic influences.

**Methods:**

A Swedish nationwide register-based cohort study was conducted to disentangle the influence of prenatal androgen exposure from familial confounding in the association between maternal PCOS and offspring attention-deficit/hyperactivity disorder (ADHD), autism spectrum disorders (ASD), and Tourette's disorder and chronic tic disorders (TD/CTD). PCOS-exposed offspring (*n* = 21 280) were compared with unrelated PCOS-unexposed offspring (*n* = 200 816) and PCOS-unexposed cousins (*n* = 17 295). Associations were estimated with stratified Cox regression models.

**Results:**

PCOS-exposed offspring had increased risk of being diagnosed with ADHD, ASD, and TD/CTD compared with unrelated PCOS-unexposed offspring. Associations were stronger in girls for ADHD and ASD but not TD/CTD [ADHD: adjusted hazard ratio (aHR) = 1.61 (95% confidence interval (CI) 1.31–1.99), ASD: aHR = 2.02 (95% CI 1.45–2.82)] than boys [ADHD: aHR = 1.37 (95% CI 1.19–1.57), ASD: aHR = 1.46 (95% CI 1.21–1.76)]. For ADHD and ASD, aHRs for girls were stronger when compared with PCOS-unexposed cousins, but slightly attenuated for boys.

**Conclusions:**

Estimates were similar when accounting for familial confounding (i.e. genetics and environmental factors shared by cousins) and stronger in girls for ADHD and ASD, potentially indicating a differential influence of prenatal androgen exposure *v.* genetic factors. These results strengthen evidence for a potential causal influence of prenatal androgen exposure on the development of male-predominant neuropsychiatric disorders in female offspring of women with PCOS.

## Introduction

Prenatal androgen exposure has been hypothesized to be associated with the development of neuropsychiatric disorders including autism spectrum disorders (ASD) (Baron-Cohen *et al*., 2011), attention-deficit/hyperactivity disorder (ADHD) (de Bruin *et al*., 2006; Baron-Cohen *et al*., 2011), and Tourette's disorder and chronic tic disorders (TD/CTD) (Peterson *et al*., 1992; Alexander and Peterson, 2004; Martino *et al*., 2013). Additionally, some psychiatric disorders show a response to treatment with anti-androgens, such as TD/CTD (Peterson *et al*., 1994, 1998), potentially implicating altered androgenic pathways in their etiology.

Polycystic ovary syndrome (PCOS) is the most common endocrine disorder affecting women of reproductive age (Rotterdam ESHRE/ASRM-Sponsored PCOS Consensus Workshop Group, 2004). The most prominent feature of PCOS is hyperandrogenism, characterized by elevated testosterone levels in serum and an increased testosterone to sex hormone-binding globulin ratio, which persists throughout a woman's reproductive years (Goodarzi *et al*., 2011). Previous studies have reported significantly higher levels of androgens throughout the pregnancies of women with PCOS, compared with pregnancies of women without PCOS (Sir-Petermann *et al*., 2002; Anderson *et al*., 2010; Barry *et al*., 2010; Maliqueo *et al*., 2013).

Studies have therefore utilized maternal PCOS as a model of offspring exposure to elevated levels of prenatal androgens, reporting that the children of women with PCOS are at increased risk of both ASD and ADHD (Palomba *et al*., 2012; Kosidou *et al*., 2016, 2017; Cherskov *et al*., 2018).

However, women with PCOS themselves have an elevated prevalence of a range of psychiatric disorders compared with women without PCOS, including ASD and ADHD (Ingudomnukul *et al*., 2007; Herguner *et al*., 2015; Cesta *et al*., 2016; Cherskov *et al*., 2018). Because these disorders are moderate to highly heritable (Posthuma and Polderman, 2013; Mataix-Cols *et al*., 2015), it is possible that the association between maternal PCOS and offspring neuropsychiatric disorders is confounded by shared genetic influences. Furthermore, some familial environmental factors associated with having received a diagnosis of maternal PCOS (e.g. access to health care, healthcare seeking behavior) may also influence whether the child receives a neuropsychiatric diagnosis and therefore may confound the association. Hence, further investigation is needed to disaggregate the influence of prenatal androgen exposure from familial confounding (i.e. genetic and/or environmental factors).

The first aim of this study was to measure, in the general population, the association between maternal PCOS and offspring neuropsychiatric disorders where prenatal androgen levels and/or altered androgen function have been implicated in their etiology. The second aim was to determine whether these associations were sensitive to familial confounding by comparing the risk of these same neuropsychiatric disorders in the offspring of siblings of women with PCOS. To this end, we compared cousins who differ in their prenatal androgen exposure, but share 12.5% of segregated genes and environmental factors shared by cousins that make them similar (D'Onofrio *et al*., 2013). If the increased risk for psychiatric disorders in the offspring of women with PCOS is mainly driven by familial factors, we would expect attenuated hazard ratios (HR) in the analysis which account for familial factors. However, if familial factors are not driving the findings (i.e. indicating a possible causal effect of prenatal androgen exposure), then the risk estimates for neuropsychiatric disorders should be similar when PCOS-exposed offspring are compared with PCOS-unexposed unrelated and related offspring.

Notably, rodent models of prenatal androgen exposure have measured anxiety-like behavior in female offspring, and to a lesser extent in the male offspring, attributable to changes in hormone receptor expression in the amygdala (Hu *et al*., 2015). Therefore, the third aim was to assess if the associations in the PCOS-exposed *v.* PCOS-unexposed offspring differed by sex.

## Methods

### Data sources

Using the unique personal identification number assigned to each individual in Sweden at birth or at immigration, several nationwide longitudinal registers containing health and sociodemographic data until 31 December 2013 were linked (see online Supplementary material for more details), including the Swedish Medical Birth Register (MBR), National Patient Register (NPR), Prescribed Drug Register (PDR), Multi-Generation Register (MGR), Total Population Register (TPR), Migration Register, and the Cause of Death Register (CDR).

### Study population and exposure classification

Women who had delivered at least one child were identified through the MGR. The first diagnostic criteria for PCOS were established in 1990 (Rotterdam ESHRE/ASRM-Sponsored PCOS Consensus Workshop Group, 2004). Those women with PCOS were identified by having at least one PCOS International Classification of Diseases (ICD) code (ICD-9: 256E; ICD-10: E28.2) recorded in the MBR or in the NPR after the age of 13 and between 1990 and 2013. Women with a concurrent diagnosed condition that could cause symptoms similar to PCOS were excluded to ensure specificity (see online Supplementary Material for ICD codes). This yielded a total of 12 955 mothers with PCOS. Time of PCOS diagnosis (before or after delivery) was not taken into account, since elevated testosterone levels have been reported to be present throughout life in women with PCOS (Pinola *et al*., 2015). Each mother with PCOS was then matched on her birth year and county of residence within the year of diagnosis to 10 comparison mothers without a PCOS diagnosis randomly selected from the general population. Last, all full brothers (*n* = 4467) and sisters (*n* = 7404) of women with PCOS with children were identified from the MGR.

**Fig. 1.**
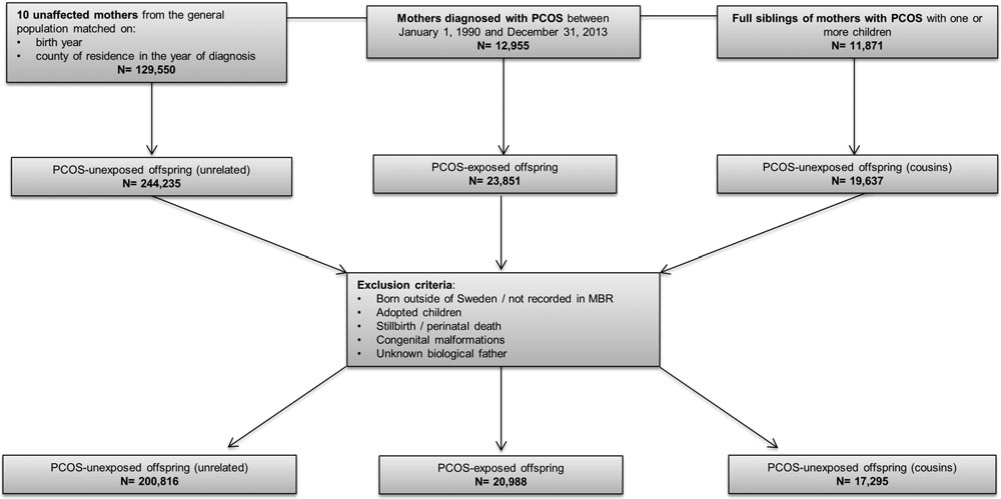
Flowchart of inclusion and exclusion criteria of study population. PCOS, polycystic ovary syndrome.

Using the MGR, offspring born to mothers with PCOS (PCOS-exposed), matched unaffected mothers (PCOS-unexposed), and full siblings were identified (PCOS-unexposed cousins). All offspring from each mother were included. Offspring were excluded if they were born outside of Sweden, adopted, stillborn or died on the day of birth, or had congenital malformations. This yielded a total of 20 988 PCOS-exposed offspring, 200 816 PCOS-unexposed offspring from the general population, and 17 295 PCOS-unexposed cousins (Fig. 1). Offspring were born between 1973 and 2013, leading to an age range of 0–40 years at the end of follow-up.

### Outcome classification

*ADHD* – Individuals with either a diagnosis of ADHD in the NPR (ICD-9: 314; ICD-10: F90) or with a filled prescription of ADHD medication in the PDR [methylphenidate (Anatomical Therapeutic Chemical [ATC] classification = N06BA04); amphetamine [ATC = N06BA01]; dexamphetamine [ATC = N06BA02]; atomoxetine [ATC = N06BA09]; or lisdexamfetamine [ATC = N06BA12]] after age 3 were considered to have a diagnosis of ADHD. The date of diagnosis was defined as the date of the first diagnosis or first prescription of ADHD medication, whichever came first (Chen *et al*., 2017).

*ASD* – Individuals were identified as having ASD if they had at least one registered code in the NPR (ICD-9: 299 or ICD-10: F84), received after the age of 1.

*TD*/*CTD* – Individuals with a diagnosis of TD/CTD after 3 years of age were identified using a previously validated algorithm based on ICD codes (ICD-9: 306.2, 307C; ICD-10: F95), shown to have excellent positive predictive value when compared with clinical records (Ruck *et al*., 2015).

### Covariates

Highest attained maternal education level was used as a proxy for offspring socioeconomic status. Maternal and paternal lifetime history of psychiatric disorders was determined by any recorded psychiatric diagnosis in the NPR. Region of birth (Nordic/non-Nordic) was extracted from the TPR. Offspring sex and year of birth, as well as maternal age at the offspring's birth, were extracted from the MBR.

### Statistical analysis

Offspring were followed from the age when they were eligible to receive a diagnosis of the respective psychiatric conditions to the date of diagnosis of the neuropsychiatric disorder of interest, death, emigration, or end of follow-up (31 December 2013), whichever came first. Associations between maternal PCOS and offspring neuropsychiatric disorders in the general population were estimated as HR with 95% confidence intervals (CI) using stratified Cox regression models with attained age as the underlying time scale. Each psychiatric disorder was modeled separately in both crude and adjusted models. In addition to the maternal matching criteria (maternal birth year and county of residence within the year of PCOS diagnosis), potential confounding was addressed in the fully adjusted models by controlling for offspring sex and year of birth, maternal age at child's birth, maternal education, maternal region of birth, and maternal and paternal lifetime history of psychiatric disorders. Robust standard errors were used to account for dependence between observations since several children from the same family were included in the study population. In order to assess if the associations remained after adjusting for familial confounding, the analyses were repeated in the sample with PCOS-unexposed cousins in a similar manner. Cousins share on average 12.5% of the segregating genes. Furthermore, some environmental risk factors may be shared between cousins as well (e.g. family background characteristics such as socioeconomic position). Therefore, comparing differentially exposed cousins controls for both measured covariates included in the model, and reduces confounding from unmeasured factors (both genetic and environmental) shared between cousins (D'Onofrio *et al*., 2013).

The analysis was conducted first for all offspring combined, and then stratified by offspring sex. We performed Wald tests to formally test if the associations statistically differed by offspring sex.

### Sensitivity analyses

*Year of PCOS diagnosis –* Diagnostic criteria for PCOS changed over the study period (Rotterdam ESHRE/ASRM-Sponsored PCOS Consensus Workshop Group, 2004). Therefore, a stratified analysis of offspring born to women with their first PCOS diagnoses between 1990 and 2003 and between 2004 and 2013 was conducted.

*Offspring from brothers v. sisters of women with PCOS –* Depending on the diagnostic criteria used, the prevalence of PCOS varies from 5% to 15% in women of reproductive age (March *et al*., 2010). Given that the prevalence of a PCOS diagnosis in the NPR is lower than expected (approximately 2%),(Cesta *et al*., 2016) and the high heritability of PCOS (60–70%) (Vink *et al*., 2006; Cesta *et al*., 2017), it is highly likely that there are sisters of women with PCOS misclassified as ‘unaffected’ because they have not received a diagnosis of PCOS. The downstream implication of this is that their offspring would be misclassified as unexposed cousins in the primary analysis. However, the risk of misclassification is lower for the offspring of brothers of women with PCOS. Therefore, we performed the analysis for the unexposed cousins born to brothers and sisters of women with PCOS separately.

*Outpatient coverage –* The NPR includes only diagnoses made in inpatient care until 2001 when outpatient specialist care was added to the register. Therefore, if an individual received a diagnosis in outpatient care before 2001, they would only be identified as a case in this study if there was a subsequent diagnosis after 2001. This will lead to either a misclassification of the outcome, or left censoring of incident cases before 2001, thereby leading to a longer time to event. Hence, a sensitivity analysis was conducted where children were included only if their outpatient care was covered in the NPR. The minimum age for diagnosis for each of the outcomes varied and therefore different years of birth were used for each analysis: the analysis for ADHD included children born from 1998, for ASD children born from 2000, and for TD/CTD from 1998.

All analyses were performed using Stata statistical software version 14.0 (Stata Corps, Texas, USA).

## Results

### Descriptive characteristics

Descriptive characteristics of the study population by category of exposure are presented in Table 1. PCOS-exposed offspring were more often born to mothers who were slightly older and who had a lifetime history of psychiatric diagnoses, compared with both categories of PCOS-unexposed offspring. ASD, ADHD, and TD/CTD were more prevalent in PCOS-exposed offspring compared with PCOS-unexposed offspring born to unrelated mothers from the general population, but prevalence estimates were similar to their PCOS-unexposed cousins. The mean age (±standard deviation) of the offspring at the end of follow-up was 9.2 ± 7.6 years.

**Table 1.** Descriptive characteristics of study population

Characteristic	PCOS-exposed offspring (*N* = 20 988)		PCOS-unexposed offspring (unrelated) (*N* = 200 816)		PCOS-unexposed offspring (cousins) (*N* = 17 295)	
	*n*	%	*n*	%	*n*	%
Sex of offspring						
Male	10 779	51.36	102 528	51.06	8940	51.69
Female	10 209	48.64	98 288	48.94	8355	48.31
Child's year of birth						
1973–1984	314	1.5	3317	1.65	817	4.72
1985–1989	539	2.57	6869	3.42	1151	6.66
1990–1994	1333	6.35	16 739	8.34	2059	11.91
1995–1999	2034	9.69	23 402	11.65	2213	12.8
2000–2004	3398	16.19	38 105	18.98	3242	18.75
2005–2009	6397	30.48	57 488	28.63	4284	24.77
2010–2013	6973	33.22	54 896	27.34	3529	20.4
Maternal age at child's birth (years)						
<20	540	2.57	6296	3.14	722	4.17
20–24	3847	18.33	43 570	21.70	4158	24.04
25–29	7108	33.87	74 121	36.91	6086	35.19
30–34	6486	30.90	57 995	28.88	4637	26.81
35+	3007	14.33	18 834	9.38	1692	9.78
Mother's region of birth						
The Nordic countries	16 595	79.07	167 284	83.30	15 432	89.23
Outside the Nordic countries	4393	20.93	33 532	16.70	1863	10.77
Maternal highest achieved education^a^						
Primary and secondary education	2152	10.3	19 650	9.85	2019	11.72
Upper secondary education	9087	43.47	88 465	44.33	8267	47.98
Post-secondary/post-graduate education	9664	46.23	91 424	45.82	6945	40.31
Maternal lifetime history of any psychiatric diagnosis	4660	22.2	31 294	15.58	2914	16.85
Paternal lifetime history of any psychiatric diagnosis	2318	11.04	20 898	10.41	1845	10.67
Offspring psychiatric disorders						
ADHD	681	3.24	5440	2.71	602	3.48
ASD	256	1.22	1765	0.88	202	1.17
TD/CTD	38	0.18	282	0.14	30	0.17

ADHD, attention-deficit/hyperactivity disorder; ASD, autism spectrum disorders; PCOS, polycystic ovary syndrome; TC/CTD, Tourette disorder/chronic tic disorder.

a Categories do not add up to the total number because maternal education is missing for 0.6% of children.

### Main analyses

**Fig. 2.**
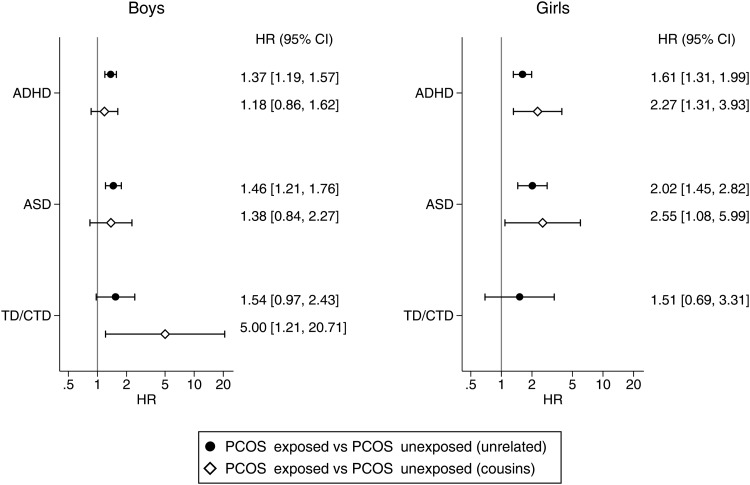
aHRs and 95% CIs for the risk of psychiatric disorders in PCOS-exposed and PCOS-unexposed offspring, stratified by sex. ADHD, attention-deficit/hyperactivity disorder; ASD, autism spectrum disorders; CI, confidence interval; HR, hazard ratio; PCOS, polycystic ovary syndrome; TD/CTD, Tourette's disorder/chronic tic disorder. Note: For the TD/CTD analyses for girls, it was not possible to fit a fully adjusted model when comparing to PCOS-unexposed cousins as the number of cases was too few.

PCOS-exposed offspring had crude HRs ranging from 1.61 to 1.90 and adjusted HRs (aHR) from 1.46 to 1.60 for being diagnosed with ADHD, ASD, or TD/CTD (Table 2), compared with PCOS-unexposed unrelated offspring. Similar estimates were found when familial factors were controlled for by comparing PCOS-exposed offspring with their unexposed cousins, albeit with wider CIs due to the smaller sample sizes (Table 2). Figure 2 and Table 2 show the HRs of the analysis stratified by offspring sex. Associations were stronger in girls than in boys for both ADHD (aHR = 1.61 *v.* aHR = 1.37) and ASD (aHR = 2.02 *v.* aHR = 1.46) when compared with PCOS-unexposed unrelated offspring from the general population. Restricting the comparison group to PCOS-unexposed cousins led to a slight attenuation of the aHRs for ADHD and ASD in boys, but a slight increase in the aHR in girls. The difference between boys and girls was statistically significant for ADHD when PCOS-exposed offspring were compared with their unexposed cousins (*p*-value: 0.038), but not for any other association.

**Table 2.** Crude and adjusted hazard ratios and 95% CIs for the risk of psychiatric disorders in PCOS-exposed and PCOS-unexposed offspring

Outcome	All			Boys			Girls		
	No. of cases	Crude	Adjusted^a^	No. of cases	Crude	Adjusted^b^	No. of cases	Crude	Adjusted^b^
	Exposed/unexposed	HR (95% CI)	HR (95% CI)	Exposed/unexposed	HR (95% CI)	HR (95% CI)	Exposed/unexposed	HR (95% CI)	HR (95% CI)
PCOS-exposed compared with PCOS-unexposed offspring (unrelated)									
ADHD	681/5440	1.61 (1.45–1.79)	1.46 (1.31–1.63)	449/3758	1.52 (1.34–1.73)	1.37 (1.19–1.57)	232/1682	1.72 (1.42–2.09)	1.61 (1.31–1.99)
ASD	256/1765	1.79 (1.53–2.09)	1.57 (1.34–1.84)	177/1297	1.61 (1.33–1.95)	1.46 (1.21–1.76)	79/468	2.29 (1.68–3.11)	2.02 (1.45–2.82)
TD/CTD	38/282	1.90 (1.30–2.78)	1.60 (1.08–2.37)	30/220	1.76 (1.12–2.75)	1.54 (0.97–2.43)	8/62	1.69 (0.76–3.73)	1.51 (0.69–3.31)
PCOS-exposed offspring compared with PCOS-unexposed offspring (cousins)									
ADHD	681/602	1.50 (1.22–1.84)	1.39 (1.10–1.76)	449/418	1.27 (0.98–1.66)	1.18 (0.86–1.62)	232/184	2.40 (1.57–3.67)	2.27 (1.31–3.93)
ASD	256/202	1.76 (1.29–2.41)	1.49 (1.06–2.10)	177/156	1.81 (1.19–2.75)	1.38 (0.84–2.27)	79/46	2.64 (1.19–5.82)	2.55 (1.08–5.99)
TD/CTD	38/30	1.91 (0.98–3.73)	1.61 (0.77–3.35)	30/23	4.31 (1.52–12.22)	5.00 (1.21–20.71)	8/7	0.92 (0.12–7.26)	N/A

ADHD, attention-deficit/hyperactivity disorder; ASD, autism spectrum disorders; CI, confidence interval; HR, hazard ratio; PCOS, polycystic ovary syndrome; TD/CTD, Tourette disorder/chronic tic disorders.

N/A = was not possible to fit the adjusted model due to too few cases.

a Model adjusted for offspring sex and year of birth, maternal age at child's birth, maternal education, maternal region of birth, and maternal and paternal lifetime history of psychiatric disorders.

b Model adjusted for offspring year of birth, maternal age at child's birth, maternal education, maternal region of birth, and maternal and paternal lifetime history of psychiatric disorders.

Risk of receiving a diagnosis for TD/CTD was similar between girls and boys (aHR = 1.51 *v.* aHR = 1.54) with overlapping CIs. When compared with PCOS-unexposed cousins, the aHR increased for boys, although with much wider CIs around the estimate. For girls, it was not possible to fit a fully adjusted model when comparing to PCOS-unexposed cousins as the number of TD/CTD cases was too few.

Kaplan–Meier curves are presented in online Supplementary Fig. S1, and Table S1 presents the follow-up time in person years among exposed and unexposed, shown separately for each outcome.

### Sensitivity analyses

*Year of PCOS diagnosis* – Of the mothers with PCOS, 25% received their first PCOS diagnosis between 1990 and 2003, and they bore 28% of the PCOS-exposed offspring in this cohort. Table 3 shows the results of the analysis stratified by the years of PCOS diagnosis for ADHD and ASD only, as the number of cases in each of the strata for TD/CTD were too few. The estimates for both time periods were largely comparable with those of the primary analysis.

**Table 3.** Adjusted hazard ratios and 95% CIs for the risk of ADHD and ASD in PCOS-exposed and PCOS-unexposed offspring, by year of maternal PCOS diagnoses

Outcome	PCOS-unexposed offspring (unrelated)		PCOS-unexposed offspring (cousins)	
	*n* = exposed/unexposed	aHR (95% CI)	*n* = exposed/unexposed	aHR (95% CI)
ADHD				
1990–2003	291/1962	1.56 (1.32–1.84)	291/234	1.35 (0.96–1.89)
2004–2013	390/3479	1.39 (1.20–1.61)	390/368	1.42 (1.03–1.95)
ASD				
1990–2003	97/661	1.62 (1.28–2.05)	97/73	1.39 (0.84–2.28)
2004–2013	159/1105	1.54 (1.24–1.90)	159/129	1.57 (1.00–2.47)

Models adjusted for offspring sex and year of birth, maternal age at child's birth, maternal education, maternal region of birth, and maternal and paternal lifetime history of psychiatric disorders.

ADHD, attention-deficit/hyperactivity disorder; ASD, autism spectrum disorders; CI, confidence interval; HR, hazard ratio; PCOS, polycystic ovary syndrome.

*Offspring of brothers v. sisters of women with PCOS* – There was no clear difference in estimates calculated separately for offspring of brothers (*n* = 8584) and sisters (*n* = 9454) of women with PCOS (online Supplementary Table S2). These results were also similar to the estimates reported in the primary analysis.

*Outpatient coverage* – Estimates calculated for children with follow-up in outpatient care were similar to those in the main analysis (online Supplementary Table S3).

## Discussion

In this national register-based study of mothers and their offspring, we found that children born to mothers with PCOS had a higher risk of being diagnosed with ADHD, ASD, and TD/CTD, compared with children born to mothers from the general population without PCOS. For ADHD and ASD, the comparison to PCOS-unexposed cousins attenuated the associations in boys and increased the estimates in girls. This supports the theory of prenatal androgen exposure influencing the development of some neuropsychiatric disorders, at least in female offspring, over and above shared familial factors.

The organizational/activational theory of steroid hormones posits that prenatal exposure to elevated androgens may cause permanent alterations (organizational effects) of neural systems leading to ‘hyper-masculine’ behavioral and cognitive traits, thereby increasing the risk for the development of neurodevelopmental and psychiatric disorders predominantly found in males such as ADHD, ASD, and TD/CTD (Arnold, 2009; Martino *et al*., 2013). For human female fetuses, levels of androgens are very low throughout gestation, whereas male fetuses have been shown to have higher levels of testosterone from approximately the 8th to 24th week of gestation. Evidence from both human and animal studies supports a larger effect of prenatal androgen exposure on cognitive and behavioral traits in females (Auyeung *et al*., 2013; Wang *et al*., 2017). Complementary to the results from our study, prenatal androgen exposure during late pregnancy has been found to increase anxiety-like behavior in female offspring, and to a lesser extent in the male offspring, in a rodent model (Hu *et al*., 2015).

Maternal PCOS has been utilized as a model of offspring exposure to elevated levels of prenatal androgens in previous studies. Consistent with the findings from our study, Kosidou and colleagues reported increased risk for ADHD and ASD in children of women with PCOS in their case–control studies [from similarly adjusted models: odds ratio (OR) = 1.42 (95% CI 1.26–1.58), OR = 1.59 (95% CI 1.34–1.88), respectively] (Kosidou *et al*., 2016, 2017). Further adjustment for obstetrical characteristics did not significantly influence their findings; however, accounting for obesity and cardiometabolic profiles in the mothers increased the estimates. It is relevant to note that our study population partially overlaps with those of Kosidou *et al*. However, the birth years of the children included in our study extend 11 years before and 2 years after their study period, yielding at least one-third more ADHD and ASD PCOS-exposed cases in our analysis. Further, while parental psychiatric history was accounted for in the studies by Kosidou *et al*., they did not attempt to further account for the influence of genetic confounding shared by PCOS and psychiatric disorders (Ingudomnukul *et al*., 2007; Herguner *et al*., 2015; Cesta *et al*., 2016), or by familial environmental factors such as health-seeking behavior. When stratified by offspring sex, the odds for having a diagnosis of ADHD was higher in females (OR = 1.53 *v.* 1.37 for male offspring), but the same for both sexes for ASD diagnoses (Kosidou *et al*., 2016, 2017).

In line with our findings, Palomba *et al*. (2012) reported a higher risk of ASD in daughters, but not in sons, of women with PCOS (Palomba *et al*., 2012). However, Cherskov *et al*. (2018) reported a higher adjusted risk of ASD in first born sons and but not daughters of women with PCOS (Cherskov *et al*., 2018).

It is difficult to draw causal conclusions based on observational studies due to the influence of unmeasured confounding. Genetically informative designs, such as family-based study designs, can be used to better account for unmeasured confounding of factors shared in families, such as genetic influences. Sibling comparison studies are often used, because comparing exposure discordant siblings allows for the adjustment for genetic and environmental factors shared between siblings (D'Onofrio *et al*., 2013). However, elevated testosterone levels are present throughout life in women with PCOS (Pinola *et al*., 2015), and therefore theoretically all of their offspring are exposed to elevated androgens in utero. This makes it impossible to conduct a sibling comparison study, where siblings need to be discordantly exposed. Therefore, we employed an ‘offspring of siblings’ study design where differentially exposed cousins were used as a comparison group to help account for unmeasured genetic and environmental factors. Our findings, based on these cousin comparisons, suggest that elevated androgens in utero influence the development of male-predominant neuropsychiatric disorders in female offspring over and above the genetic and familial factors associated with the maternal psychiatric illness that are comorbid with PCOS. This suggests that familial confounding does not play a large role in the findings of previous studies published on this topic.

However, there are some important limitations regarding the cousin design. First, the cousin comparison design requires assumptions about equal environmental influences and variability in measures across cousin types. Although this is the first study to account for familial factors in association between maternal PCOS and offspring neuropsychiatric disorders, in comparison with a sibling comparison design for example, the cousin comparison captures a smaller fraction of the potential confounding from genetic and environmental influences. The resulting residual genetic confounding could inflate our estimates of the association, and the true association between maternal PCOS and offspring psychiatric disorders might be weaker than what we observed.

On the other hand, compared with the prevalence of PCOS in the general population, PCOS is greatly underrepresented in the NPR. Therefore, misclassification of PCOS in the mothers is a possibility which consequently misclassifies the exposure status of some of the offspring, leading to a possible dilution of the measured associations. Further, this misclassification bias has the potential for a higher impact in the analysis between the PCOS-exposed offspring and their unexposed cousins, as misclassification of exposure is likely more frequent in analyses based on exposure-discordant cousins than in studies based on the general population. Due to the high heritability of PCOS, sisters of women with PCOS are expected to have higher levels of PCOS. When there is discordance between PCOS status in sisters, it is more likely to be due to misclassification than when considering women from the general population (Frisell *et al*., 2012). Therefore, we attempted to capture the degree of bias due to misclassification by analyzing cousins born to sisters of women with PCOS and cousins born to brothers of women with PCOS as two separate control groups. The results from the sensitivity analysis did not differ, indicating that it is unlikely that there is exaggerated misclassification bias in the cousin comparison analysis.

The diagnostic criteria for PCOS changed over the study period. The first diagnostic guidelines for PCOS established by the National Institute of Health in 1990 required the presence of both hyperandrogenemia and oligo- and/or anovulation for diagnosis. In 2003, the Rotterdam diagnostic guidelines were issued and introduced a third possible criterion – polycystic ovaries (Rotterdam ESHRE/ASRM-Sponsored PCOS Consensus Workshop Group, 2004). As only two of the three criteria are required for PCOS diagnosis under the latter guidelines, there are PCOS phenotypes that do not include hyperandrogenemia, thereby creating a more heterogeneous group of women with PCOS in the later study years. Therefore, by stratifying the analysis based on year of PCOS diagnosis, it is more certain that children born to women with PCOS diagnosed between 1990 and 2003 were exposed to higher prenatal androgen levels. However, findings from this stratified analysis were similar to the main analysis. This may be due to significant referral bias for PCOS, where women with more complete phenotypes of PCOS (i.e. with hyperandrogenemia) and higher body mass index are more likely to seek and receive medical care (Lizneva *et al*., 2016), leading to a more homogeneous population of women diagnosed with PCOS in all years of our study.

Additionally, daughters of women with PCOS have a high likelihood of being diagnosed with PCOS, which could account for the higher rates of psychiatric disorders in this study (Xita and Tsatsoulis, 2006). However, symptoms of PCOS do not emerge before puberty and the appearance of a diagnosis in the Swedish National Registers occurs at a mean age of 28 years (Cesta *et al*., 2016). Therefore, due to the young age of the cohort, it is not possible to identify offspring with a potential PCOS diagnosis. Further, by comparing cousins we adjusted for some of the genetic factors influencing both PCOS and psychiatric disorders.

The main strengths of this study include the use of longitudinal population-based national register data to assess the association of maternal PCOS with neuropsychiatric outcomes in the offspring, and the use of a cousin comparison group to adjust for familial confounding. Further, this is the first study to assess the risk of TD/CTD in children of mothers with PCOS, and the first to include multiple psychiatric outcomes based on clinical diagnoses.

In addition to the limitations of the cousin-design discussed above and the change in the diagnostic criteria for PCOS, we did not have actual laboratory data on the androgen status during pregnancy of mothers with PCOS in this study. As a proxy, we performed a stratified analysis based on the diagnostic criteria of PCOS, identified by the year of PCOS diagnosis.

The role of the prenatal period in the etiology of neuropsychiatric disorders in children is of considerable research and clinical interest. Prenatal exposure to androgens has been the subject of many animal, clinical, and observational studies. Due to the elevated levels of androgens that are characteristic of PCOS, maternal PCOS has recently been used as a model for measuring the risk of elevated prenatal androgen exposure on the development of offspring psychiatric disorders. However, the results of these studies are at risk of being influenced by familial confounding due to the higher prevalence of psychiatric disorders in women with PCOS. We used a unique cousin-comparison design – which accounted for some degree of unmeasured familial confounding – made feasible by the comprehensive Swedish national registers. Our results add to a growing body of evidence supporting the influence of prenatal androgen exposure on the development of male-predominant neuropsychiatric disorders in children born to women with PCOS, with potentially differential influence of the prenatal environment *v.* genetics depending on the sex of the offspring. However, due to the limitations of the cousin design, these findings should be confirmed using other approaches and study designs.

## Supplementary material

For supplementary material accompanying this paper visit http://dx.doi.org/10.1017/S0033291719000424. click here to view supplementary material
