# Supplementary material for: Maternal polycystic ovary syndrome and risk of neuropsychiatric disorders in offspring: prenatal androgen exposure or genetic confounding?
Source: Psychol Med. 2019 Mar 12;50(4):616–24. doi: 10.1017/S0033291719000424 (PMC7093321; doi:10.1017/S0033291719000424)

**Supplementary Material**

Maternal Polycystic Ovary Syndrome and Risk of Neuropsychiatric Disorders in Offspring: In Utero Androgen Exposure or Genetic Confounding?

Carolyn E. Cesta*, Anna S Öberg, Abraham Ibrahimson, Ikram Yusuf, Henrik Larsson, Catarina Almqvist, Brian D’Onofrio, Cynthia M. Bulik, Lorena Fernández de la Cruz, David Mataix-Cols, Mikael Landén, Mina A. Rosenqvist.

*Corresponding author

Affiliation: Karolinska Institutet

carolyn.cesta@ki.se

**Supplementary Methods**

1. **Additional information on the Swedish National Registers**

A unique personal identity number (PIN) is issued to all citizens of Sweden upon birth, immigration, or to all residents staying at least 1 year in Sweden, and is used unchanged throughout life. The PIN is used to link patient-level data from the different registers (Ludvigsson *et al*, 2009). All residents and citizens, independent of socioeconomic status, have unrestricted access to health services including partial or complete reimbursement of purchased medicines because of a tax-supported public health service with universal coverage. The **Total Population Register** (Ludvigsson *et al*, 2016) includes data on demographic variables for all Swedish residents including information on migration, death and education, and can be linked to the major national healthcare registers in Sweden, including:

The **Prescribed Drug Register** (PDR) (Wettermark *et al*, 2007; Furu *et al*, 2010; Wettermark *et al*, 2013) includes information on all prescribed drugs dispensed for the entire Swedish population since July 1, 2005. The register contains patient level data on dispensed medicine including information on the dispensed drug (product, quantity, price) as well as the dates of prescription and dispensing. Data on total and reimbursed expenditure and certain characteristics of the prescriber and the workplace of the prescriber are also recorded. All drugs are classified according to the World Health Organization’s Anatomical Therapeutic Chemical Classification System (ATC) codes and the register is updated monthly. Medications administered during hospitalization are not available in the PDR.

The Swedish **National Patient Register** (NPR) (Ludvigsson *et al*, 2011) includes more than 99% of all somatic (including surgery) and psychiatric hospital discharges and visits. It is mandatory for all physicians providing private or publicly funded hospital-based care to deliver data to the NPR. Since 1997 ICD-10 codes have been used. A Swedish version of the Nordic Medico-Statistical Committee (NOMESCO) Classification of Surgical Procedures has been used since 1997. Current procedures are listed in the Nordic Classification of Surgical and Medical Procedures (NCSP), which has also been used since 1997. The NPR was initiated by the National Board of Health and Welfare in the 1960’s when it started to collect data on individuals receiving inpatient care at public hospitals. Since 1987, the Patient Register has covered all public inpatient care in Sweden and since 2002 there is almost full coverage of all out-patient hospital visits.

The **Swedish Medical Birth Register** (MBR) (Odlind *et al*, 2003; Stephansson *et al*, 2011) was established in 1973 by an act of the Swedish Parliament, for the purpose of compiling information on ante- and perinatal factors and their importance for infant health. The basic structure of the register has remained unchanged since 1973. Pre-specified data is extracted from the records and forwarded electronically to the National Board of Health. Specific diagnoses are noted with the currently used version of ICD (International Classification of Diseases) and certain other conditions are captured by means of check boxes. Since 1995 drugs used during pregnancy are included in the birth register. The information mainly includes drugs used in early pregnancy and is based on the information provided by the pregnant woman at the first visit to antenatal care.

The **Cause of Death Register** (CDR) (Brooke *et al*, 2017) comprises all deaths among Swedish residents, whether occurring in Sweden or abroad. The causes of death are coded centrally at Statistics Sweden according to the international (English) version of ICD-10. The NPR can be linked and matched with the cause-of-death register to attain an even better coverage of disease events and, to some extent, to include patients managed outside hospitals.

The **Multi-Generation Register** (MGR) (Ekbom, 2011) links all individuals (index persons) born since 1932 and alive in 1961 to their biological and adoptive parents. From 1961 onward, the register has excellent coverage with information on 100% of the biological mothers, and 98% of fathers of all individuals born in Sweden. The coverage is less complete for index persons born outside Sweden as information on their parents is only included if they immigrated before the age of 18 years. The link between children and parents makes it possible to construct large pedigrees of different family relationships: full and half-siblings, aunts/uncles, cousins, and even grandparents.

**The Database for Health Insurance and Labor Market** (LISA) is a longitudinal integrated database and since 1990 contains information from the labor market, educational and social sectors for all individuals registered in Sweden who are 16 years of age and older.

**References**

**Brooke, HL, Talback, M, Hornblad, J, Johansson, LA, Ludvigsson, JF, Druid, H, Feychting, M and Ljung, R** (2017) The Swedish cause of death register. *European journal of epidemiology*.

**Ekbom, A** (2011) The Swedish Multi-generation Register. *Methods in Molecular Biology* **675**, 215-220.

**Furu, K, Wettermark, B, Andersen, M, Martikainen, JE, Almarsdottir, AB and Sorensen, HT** (2010) The Nordic countries as a cohort for pharmacoepidemiological research. *Basic & Clinical Pharmacology & Toxicology* **106**, 86-94.

**Ludvigsson, JF, Almqvist, C, Bonamy, AK, Ljung, R, Michaelsson, K, Neovius, M, Stephansson, O and Ye, W** (2016) Registers of the Swedish total population and their use in medical research. *European journal of epidemiology* **31**, 125-136.

**Ludvigsson, JF, Andersson, E, Ekbom, A, Feychting, M, Kim, JL, Reuterwall, C, Heurgren, M and Olausson, PO** (2011) External review and validation of the Swedish national inpatient register. *BMC public health* **11**, 450.

**Ludvigsson, JF, Otterblad-Olausson, P, Pettersson, BU and Ekbom, A** (2009) The Swedish personal identity number: possibilities and pitfalls in healthcare and medical research. *European journal of epidemiology* **24**, 659-667.

**Odlind, V, Haglund, B, Pakkanen, M and Otterblad Olausson, P** (2003) Deliveries, mothers and newborn infants in Sweden, 1973-2000. Trends in obstetrics as reported to the Swedish Medical Birth Register. *Acta Obstetricia et Gynecologica Scandinavica* **82**, 516-528.

**Stephansson, O, Granath, F, Svensson, T, Haglund, B, Ekbom, A and Kieler, H** (2011) Drug use during pregnancy in Sweden - assessed by the Prescribed Drug Register and the Medical Birth Register. *Clin Epidemiol* **3**, 43-50.

**Wettermark, B, Hammar, N, Fored, CM, Leimanis, A, Otterblad Olausson, P, Bergman, U, Persson, I, Sundstrom, A, Westerholm, B and Rosen, M** (2007) The new Swedish Prescribed Drug Register--opportunities for pharmacoepidemiological research and experience from the first six months. *Pharmacoepidemiol Drug Saf* **16**, 726-735.

**Wettermark, B, Zoega, H, Furu, K, Korhonen, M, Hallas, J, Norgaard, M, Almarsdottir, A, Andersen, M, Andersson Sundell, K, Bergman, U, Helin-Salmivaara, A, Hoffmann, M, Kieler, H, Martikainen, J, Mortensen, M, Petzold, M, Wallach-Kildemoes, H, Wallin, C and Sorensen, H** (2013) The Nordic prescription databases as a resource for pharmacoepidemiological research--a literature review. *Pharmacoepidemiology and Drug Safety* **22**, 691-699.

**PCOS Identification: Exclusion ICD codes**

Women with a concurrent diagnosed condition that could cause symptoms similar to PCOS were excluded to ensure specificity, including pituitary adenoma (ICD-8: 226.20; ICD-9: 227D; ICD-10: D352), disorders of the pituitary glands including hypo/hyperfunction (ICD-8: 253; ICD-9: 253; ICD-10: E22), disorders of the adrenal glands including congenital adrenal hyperplasia and Cushing’s syndrome (ICD-8: 255; ICD-9: 255; ICD-10: E24/E25/E27), galactorrhea (ICD-9: 611G; ICD-10: N64.3), suprarenal tumor (ICD-10: C74), or Turner’s syndrome (ICD-8: 759.5; ICD-9: 758G; ICD-10: O96).

**Supplementary Tables**

**Supplementary Table 1:** Follow-up time in person years among exposed and unexposed, shown separately for each outcome

| **Outcome** | **PCOS-exposed** | **PCOS-unexposed (unrelated)** | **PCOS-unexposed offspring (cousins)** |
| --- | --- | --- | --- |
| ADHD | 173,371 | 1,898,278 | 203,762 |
| ASD | 174,636 | 1,908,505 | 204,975 |
| TD/CTD | 175,333 | 1,913,852 | 205,601 |

**Supplementary Table 2: Crude and adjusted hazard ratios and 95% confidence intervals for the risk of psychiatric disorders in PCOS-exposed offspring compared to PCOS-unexposed cousins by PCOS sibling sex**

|  | **Crude** | **Adjusted ^a^** |
| --- | --- | --- |
| **Outcome** | **HR [95% CI]** | **HR [95% CI]** |
| **PCOS-exposed offspring compared to PCOS-unexposed cousins**  **born to *brothers* of women with PCOS (n= 8584)** | | |
| ADHD | 1.41 [1.06, 1.86] | 1.45 [1.06, 1.99] |
| ASD | 1.84 [1.20, 2.82] | 1.64 [1.01, 2.66] |
| TD/CTD | 1.88 [0.84, 4.22 | 2.24 [0.95, 5.26] |
| **PCOS-exposed offspring compared to PCOS-unexposed cousins**  **born to *sisters* of women with PCOS (n= 9454)** | | |
| ADHD | 1.69 [1.29, 2.21] | 1.54 [1.12, 2.12] |
| ASD | 1.84 [1.22, 2.77 | 1.67 [1.01, 2.77] |
| TD/CTD | 1.82 [0.75, 4.42] | 3.45 [1.35, 8.81] |

**^a^** Model adjusted for offspring sex and year of birth, maternal age at child’s birth, maternal education, maternal region of birth, and maternal and paternal lifetime history of psychiatric disorders.

Abbreviations: ADHD = attention-deficit/hyperactivity disorder; ASD = autism spectrum disorders; CI = confidence interval; HR = hazard ratio; PCOS = polycystic ovary syndrome; TD/CTD = Tourette Disorder/chronic tic disorders.

**Supplementary Table 3: Crude and adjusted hazard ratios and 95% confidence intervals for the risk of** **psychiatric disorders in PCOS-exposed offspring compared to PCOS-unexposed cousins, restricted to children covered by the outpatient register to account for left censoring of data**

|  | **Crude** | **Adjusted ^a^** |
| --- | --- | --- |
| **Outcome** | **HR [95% CI]** | **HR [95% CI]** |
| **PCOS-exposed compared to PCOS-unexposed offspring**  **(unrelated)** | | |
| ADHD | 1.64 [1.43, 1.88] | 1.58 [1.37, 1.83] |
| ASD | 1.61 [1.29, 2.01] | 1.45 [1.16, 1.83] |
| TD/CTD | 1.73 [1.05, 2.87] | 1.57 [0.92, 2.69] |
| **PCOS-exposed offspring compared to PCOS-unexposed offspring**  **(cousins)** | | |
| ADHD | 1.37[1.01, 1.84] | 1.16 [0.81, 1.66] |
| ASD | 1.77 [1.04, 3.02] | 1.56 [0.85, 2.87] |
| TD/CTD | 2.11 [0.48, 9.20] | 3.04 [0.45, 20.74]^b^ |

**^a^** Model adjusted for offspring sex and year of birth, maternal age at child’s birth, maternal education, maternal region of birth, and maternal and paternal lifetime history of psychiatric disorders.

^b^ Model adjusted for offspring sex and year of birth, maternal age at child’s birth, maternal region of birth, and maternal and paternal lifetime history of psychiatric disorders. Due to the small number of exposed cases, maternal education was omitted in order to fit a model

Abbreviations: ADHD = attention-deficit/hyperactivity disorder; ASD = autism spectrum disorders; CI = confidence interval; HR = hazard ratio; PCOS = polycystic ovary syndrome; TD/CTD = Tourette Disorder/chronic tic disorders.

**Supplementary Figure 1. Kaplan-Meier survival estimates for each outcome, shown separately for PCOS-exposed offspring, PCOS-unexposed offspring (unrelated and related)**


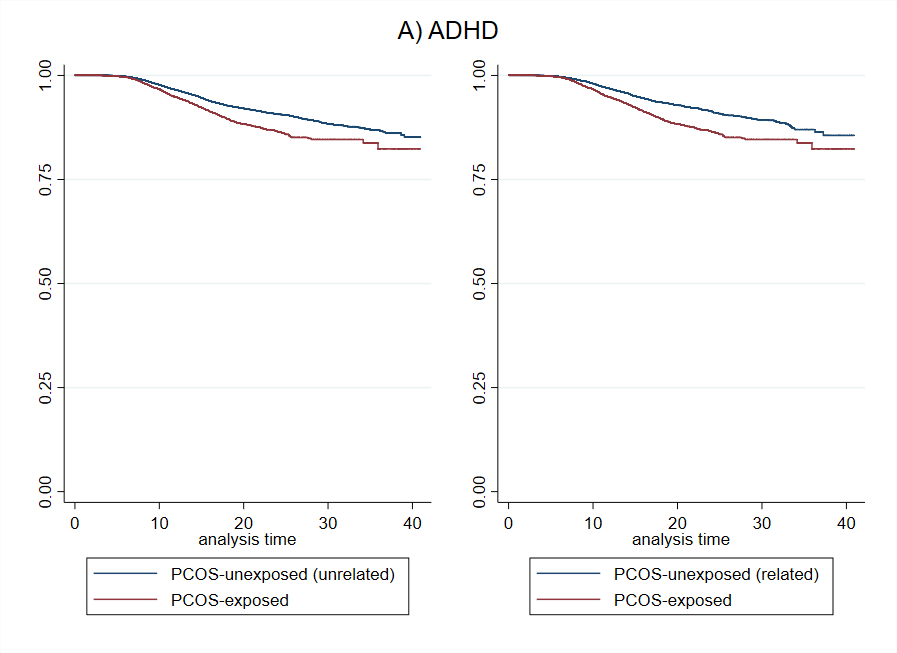


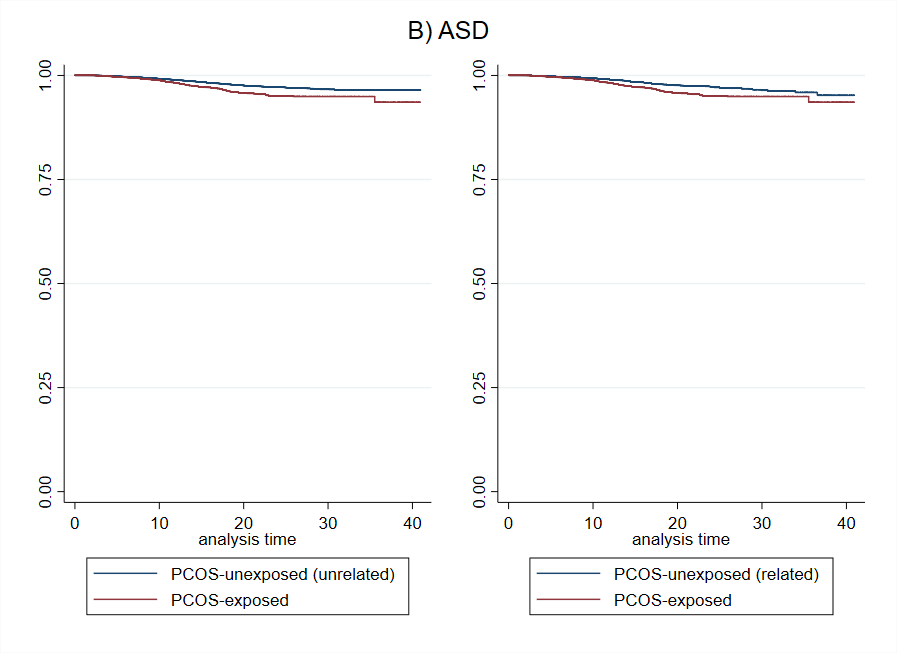
0


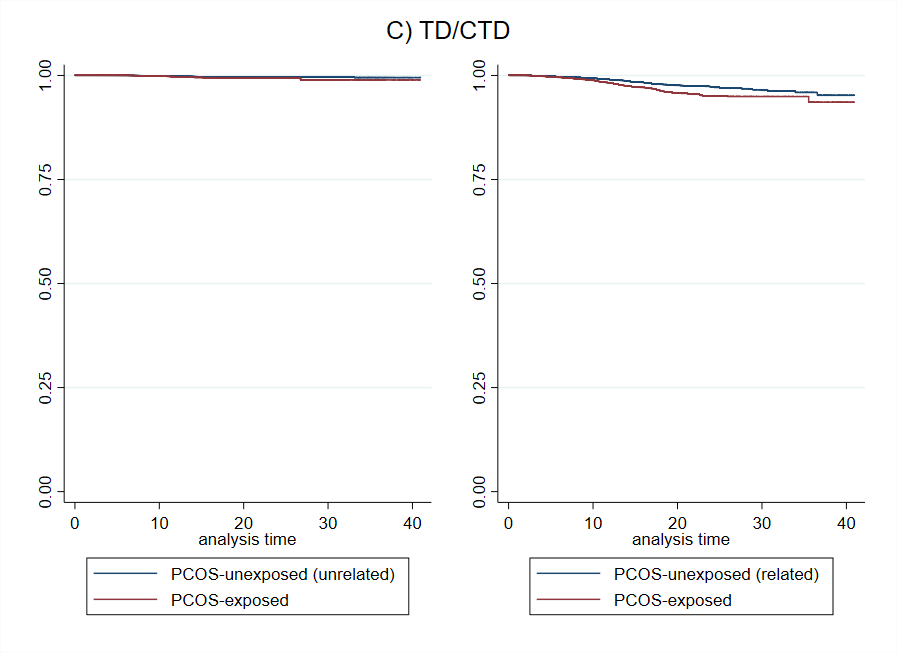

Supplement: Supplementary file 1 [file S0033291719000424sup001.docx]
